# Interpretable multimodal machine learning for injury risk stratification in adolescent athletes: integrating kinematic, physiological, and load data with SHAP/LIME explainability

**DOI:** 10.3389/fmed.2026.1848064

**Published:** 2026-07-30

**Authors:** Linzhen Zhou, Meng Zheng, Hongbing Wang, Linjun Liu, Tianshi Wei

**Affiliations:** 1 School of Physical Education, Hunan University of Technology, Zhuzhou, Hunan, China; 2 School of Sports Science and Physical Education, Nanjing Normal University, Nanjing, Jiangsu, China; 3 Faculty of Pharmacy, Lincoln University College, Petaling Jaya, Selangor, Malaysia; 4 Department of Big Data, Warsaw School of Economics, Warsaw, Poland

**Keywords:** adolescent athletes, biomechanics, explainable artificial intelligence, heart rate variability, injury risk stratification, lime, logistic regression, machine learning

## Abstract

Adolescent athletes (aged 13–18 years) face significantly elevated lower-limb injury risk compared to adult populations due to immature neuromuscular control, while existing injury risk assessment methods are broadly limited by single-modal reliance, insufficient model interpretability, and a lack of dedicated research targeting youth populations. This study proposes an interpretable multimodal machine learning framework that integrates three data modalities—kinematics, physiology, and training load—via feature-level concatenation, based on a cross-sectional multimodal dataset of 320 fully anonymised adolescent athletes comprising 12 feature variables acquired under standardized protocols using internationally recognized instruments, including Vicon optical motion capture, AMTI force platforms, Polar H10 heart rate monitors, Supersonic Imagine Aixplorer ultrasound elastography, and Catapult GPS trackers. Through 5-fold stratified cross-validation, the comprehensive performance of five traditional and five modern/deep machine learning models was systematically compared in a binary injury risk stratification task. Experimental results demonstrate that logistic regression achieved the best overall performance [Accuracy = 86.9%, AUC-ROC = 0.965, F1 = 0.866, Matthews Correlation Coefficient (MCC) = 0.742]. Dual interpretability analysis was further conducted on the optimal model using a SHapley Additive exPlanations (SHAP) linear explainer and a Local Interpretable Model-Agnostic Explanations (LIME) local explainer; both methods consistently identified peak hip adduction angle (mean |SHAP| = 2.208), ankle dorsiflexion range of motion (1.426), and dynamic knee valgus angle (1.359) as the most critical injury risk predictors (Spearman ρ = 0.93), in high agreement with established biomechanical findings in sports medicine. An ablation analysis of modality contributions demonstrated that the tri-modal integration achieved a substantive AUC gain over single-modality configurations, supporting the value of multimodal feature integration. We note explicitly that the binary risk label was derived from a clinically-informed weighted composite of the input features rather than from prospective injury follow-up; the framework therefore performs risk stratification with respect to this composite, and validation against true prospective injury outcomes remains an important next step. This study provides an interpretable technical framework for standardized, digitized, and stratified injury risk assessment in adolescent athletes, with important potential for clinical translation and sports injury prevention practice.

## 1 Introduction

Adolescent athletes (aged 13–18 years) are at a critical stage of rapid skeletal and muscular development, during which incomplete neuromuscular maturation renders the lower limb joints highly susceptible to injury during contact sports and high-intensity training (1). Epidemiological evidence indicates that anterior cruciate ligament (ACL) injury is among the most serious sports injuries in the adolescent athletic population, not only causing training interruptions lasting 6–12 months but also being strongly associated with an increased risk of cartilage degeneration and early-onset osteoarthritis (2, 3). Peak knee valgus moment, as a core biomechanical indicator of ACL injury, has been demonstrated to effectively predict athlete injury risk (2, 4), while hip adduction angle, restricted ankle dorsiflexion, training load imbalance, and autonomic nervous system dysregulation collectively constitute the complex causal system underlying injury risk in adolescent athletes (5–7).

Meanwhile, with the maturation of technologies such as Vicon motion capture, AMTI force platforms, wearable GPS sensors, and ultrasound elastography, large-scale acquisition of multimodal sports medicine data has become feasible, laying a solid data foundation for the construction of objective and precise injury risk stratification systems (8). Against this backdrop, how to effectively integrate kinematic, physiological, and training load data across heterogeneous modalities into a unified, interpretable risk-stratification model has emerged as a core scientific challenge in the field of sports injury prevention.

However, existing research continues to exhibit three systematic shortcomings in addressing these challenges. First, the informational limitations of single-modal assessment. Most existing injury risk-stratification models focus on a single data dimension—either relying exclusively on three-dimensional biomechanical indicators (2, 4) or solely on training load monitoring data (5)—with few studies systematically integrating kinematic, physiological, and load modalities. Yet injury risk formation is fundamentally the result of dynamic interactions among multidimensional factors (8); single-modal assessment inevitably fails to capture cross-modality synergistic effects, constraining stratification capability. Second, a severe lack of model interpretability. Existing studies predominantly optimize classification accuracy as the sole objective, overlooking the transparency requirements of model decision-making processes for clinicians and coaches (9, 10). Black-box models are unable to explain to practitioners “why a given athlete is classified as high risk,” thereby restricting the adoption and application of stratification systems in real-world injury intervention scenarios (11–13). Third, a critical shortage of dedicated research targeting adolescent populations. Subjects in existing machine learning injury risk-stratification studies are predominantly adult professional athletes (14–16); adolescents differ fundamentally from adults in skeletal development, neuromuscular maturity, and training adaptability, and direct transfer of adult models introduces significant physiological developmental bias.

These three shortcomings converge on a central question: *Is it possible to construct an interpretable multimodal injury risk stratification framework that is specifically designed for adolescent athletes?* To systematically address this question, the present study makes the following contributions:

1. Construction of a standardized multimodal dataset: Leveraging internationally standardized sports medicine acquisition protocols and integrating Vicon optical motion capture, AMTI force platforms, Polar H10 heart rate monitors, Supersonic Imagine Aixplorer ultrasound elastography, and Catapult GPS trackers, a multimodal injury risk dataset covering 320 adolescent athletes (aged 13–18) with 12 multimodal feature variables was constructed, filling the gap in dedicated datasets for the youth population.
2. Systematic multi-model comparison and optimal model identification: Within a unified experimental framework, ten mainstream machine learning algorithms (five traditional models and five modern/deep learning models) were compared through 5-fold stratified cross-validation, with performance comprehensively evaluated using six metrics [ACC, AUC-ROC, F1, Precision, Recall, Matthews Correlation Coefficient (MCC)], ultimately establishing logistic regression as the optimal comprehensive stratification model (AUC = 0.965, ACC = 86.9%) (17– 20).
3. A dual interpretability analysis framework with explicit acknowledgment of method-specific limitations: In the context of adolescent athlete injury risk stratification, a SHapley Additive exPlanations (SHAP) linear explainer and a Local Interpretable Model-Agnostic Explanations (LIME) local explainer were simultaneously employed to reveal key risk factors from two complementary perspectives—global feature importance ranking and local sample contribution decomposition—providing transparent and clinically-actionable communication of feature contributions; we further discuss the methodological caveat that for linear models the agreement between SHAP and LIME is partially mathematically expected, and accordingly report SHAP/LIME on a non-linear comparison model (MLP) to corroborate the feature importance ranking from an independent perspective (11–13, 21, 22).

The remainder of this paper is organized as follows. Section 2 systematically reviews related work in three directions: sports injury risk stratification, multimodal data integration, and explainable machine learning. Section 3 presents the dataset construction, preprocessing pipeline, model framework, evaluation metrics, and interpretability analysis methods in detail. Section 4 reports complete experimental results covering feature correlation analysis, multi-model performance comparison, modality ablation analysis, and SHAP/LIME interpretability analysis. Section 5 provides an in-depth discussion from four dimensions: clinical significance, rationality of model selection, methodological caveats of the label construction and explainer choice, and research limitations. Section 6 summarizes the main conclusions and outlines future research directions.

## 2 Related work

### 2.1 Sports injury risk Stratification and Prediction

Research on sports injury risk stratification has undergone a three-stage evolution from qualitative clinical screening to quantitative biomechanical modeling and subsequently to data-driven machine learning. Hewett et al. (2) pioneered a biomechanical risk-stratification framework for ACL injury based on three-dimensional motion analysis, demonstrating that peak knee valgus moment has significant predictive value for ACL injury in female athletes (sensitivity 78%, specificity 73%); Dempsey et al. (4) further revealed the effectiveness of movement control training interventions in reducing injury risk scores and knee joint biomechanics. At the etiological model level, Meeuwisse et al. (7) proposed a dynamic etiological model of sports injury emphasizing the recursive interaction of internal and external risk factors over time, providing an important theoretical framework for multifactorial comprehensive assessment; Bittencourt et al. (8) introduced a complex systems perspective, noting that traditional linear risk factor analysis cannot capture the nonlinear, multidimensional causes of injury, fundamentally advancing the application of machine learning methods in this domain.

Regarding the training load dimension, Gabbett's (5) “training-injury prevention paradox” theory systematically demonstrated the independent stratification role of the acute-to-chronic workload ratio (ACWR) for injury risk, establishing the theoretical basis for incorporating load monitoring data into injury risk models. In terms of machine learning algorithm applications, ensemble methods such as random forests (16), XGBoost (14), LightGBM (15), and CatBoost (23) have been widely adopted for their powerful capacity to handle high-dimensional nonlinear features; Soofi et al. (17) conducted a systematic review of machine learning classification techniques, noting that logistic regression can often match or surpass complex ensemble methods when features exhibit significant linear separability. Nevertheless, the above studies predominantly focus on adult professional athletes, dedicated stratification research for adolescents aged 13–18 remains scarce, and model clinical interpretability is broadly insufficient. Yuan et al. (20) demonstrated an effective framework combining ensemble learning with SHAP interpretation in the domain of cardiovascular risk prediction, providing an important methodological reference for the present study.

### 2.2 Multimodal data integration

The multidimensional etiology of sports injury risk necessitates multimodal data integration. Single-modal data—whether purely kinematic indicators (2, 4), purely physiological monitoring data (6, 24), or training load records alone (5)—are all unable to fully reflect the complex interactive causes of injury risk (7, 8). The low-frequency-to-high-frequency power ratio of heart rate variability (HRV) serves as an effective quantitative indicator of autonomic nervous system activity (6) and has been demonstrated to be closely associated with exercise fatigue status and injury susceptibility (24–26); Fooken (24) confirmed that HRV indicators can effectively capture changes in physiological arousal during high-intensity decision-making scenarios, providing cross-domain evidence for the value of physiological monitoring data.

Regarding multimodal feature integration, Xu et al. (27) proposed a dynamic multimodal feature recognition framework that achieved effective fusion of heterogeneous data in mental health assessment, while Joshi et al. (28) validated the significant gain of cross-modality information complementarity in a multimodal fusion detection task combining behavioral and physiological data. Two principal paradigms can be distinguished. The first is feature-level concatenation, in which heterogeneous modality-specific features are standardized and joined into a single vector before being passed to a downstream classifier; this paradigm is simple, sample-efficient, and well-suited to structured tabular inputs of modest dimensionality. The second is attention-based cross-modal fusion, in which the model learns adaptive inter-modal weights and explicitly captures non-linear modality interactions. The attention mechanism (29), as a core technology in deep learning, achieves dynamic information aggregation by adaptively learning correlation weights among different features or modalities; cross-modal attention further extends this capability across different data sources, enabling models to dynamically identify the relative importance of each modality during inference. The cross-attention fusion mechanism within the multi-scale temporal deep learning framework proposed by Liu et al. (30) provides an important architectural reference for multimodal integration, and the decentralized neural framework demonstrated by Yang et al. (31) in consumer IoT scenarios further validates the broad applicability of multi-source data fusion in intelligent monitoring systems.

More recently, Rahman et al. (32) proposed a fine-grained attention and geometric correspondence model for musculoskeletal risk classification in athletes, integrating multimodal visual and skeletal features and demonstrating the strong potential of attention-based architectures in sports-injury-related risk modeling on high-dimensional perceptual data. Such attention-based frameworks are particularly well-suited to large-scale visual–skeletal inputs with rich non-linear cross-modal interactions. In contrast, the present study operates on a structured tabular feature space of 12 standardized biomechanical, physiological, and load indicators on a modest cohort (*n* = 320), a regime in which lightweight linear and tree-based models tend to be more sample-efficient and clinically interpretable than deep attention models. We therefore adopt feature-level multimodal integration paired with logistic regression as the primary stratification model, and position attention-based deep cross-modal fusion as an important direction for future work on larger-scale field cohorts (see Section 5.3).

### 2.3 Explainable machine learning

As machine learning models have been more deeply applied in medical and sports science domains, model interpretability has emerged as a core bottleneck for clinical translation. Burkart and Huber (9) systematically reviewed interpretability methods for supervised learning, identifying model transparency as a key factor influencing the trustworthiness and clinical adoption rate of medical decision systems; Dwivedi et al. (10) further categorized the core technologies of explainable AI into three paradigms: *post-hoc* explanation, intrinsic transparency, and conversational explanation. SHAP (SHapley Additive exPlanations) (21), grounded in the game-theoretic Shapley value principle, provides theoretically rigorous unified contribution quantification for each feature on each prediction sample, satisfying three axiomatic constraints: local accuracy, consistency, and missingness (22); Lundberg et al. (22) further proposed the TreeSHAP algorithm for tree ensemble models, reducing computational complexity from exponential to polynomial. LIME (Local Interpretable Model-Agnostic Explanations) (11) provides intuitive conditional rule-based local explanations by constructing weighted linear approximation models in the local neighborhood of prediction samples, and is applicable to arbitrary black-box classifiers.

Salih et al. (12) conducted a systematic comparative evaluation of SHAP and LIME, indicating that the consistency of feature importance rankings between the two methods is an important standard for assessing explanation robustness on non-linear models; the practical SHAP application in drug development by Ponce-Bobadilla et al. (13) and the improved SHAP explanation framework by Nohara et al. (33) both confirm the high practical utility of this method in biomedical domains. It is important to note, however, that for an inherently linear classifier such as logistic regression, SHAP values from a linear explainer are mathematically proportional to the product of the standardized feature value and the model coefficient, and LIME's locally weighted linear surrogate will recover essentially the same structure; consequently, a high SHAP–LIME ranking agreement on a linear model is partially expected by construction rather than constituting independent cross-method validation. This consideration motivates our complementary application of SHAP/LIME to a non-linear comparison model (MLP) in Section 4. The research paradigm of Meng et al. (34), who simultaneously applied SHAP analysis to reveal key predictors in personality trait classification, is highly consistent with the interpretability analysis framework of the present study.

The comparative study of multiple explainable machine learning methods in travel mode choice prediction by Tamim Kashifi et al. (35), the multivariate outcome machine learning inference framework by Benkeser et al. (36), and the work of Zhang et al. (37) combining graph neural networks with interpretability analysis for medical prediction all provide cross-domain methodological references for the method design of this study. The systematic approach to model decision trustworthiness within the perceptual uncertainty quantification framework proposed by Tian et al. (38) further inspired the design of the dual-explainer cross-validation mechanism in this study. In summary, explainable machine learning has established a relatively complete theoretical and methodological foundation; however, the systematic application of a dual SHAP-and-LIME framework—with explicit treatment of the linear-model identifiability caveat—to injury risk stratification in adolescent athletes currently remains an unexplored area, and the present study makes a pioneering contribution in this direction.

## 3 Methods

### 3.1 Overall framework

**Figure 1:**
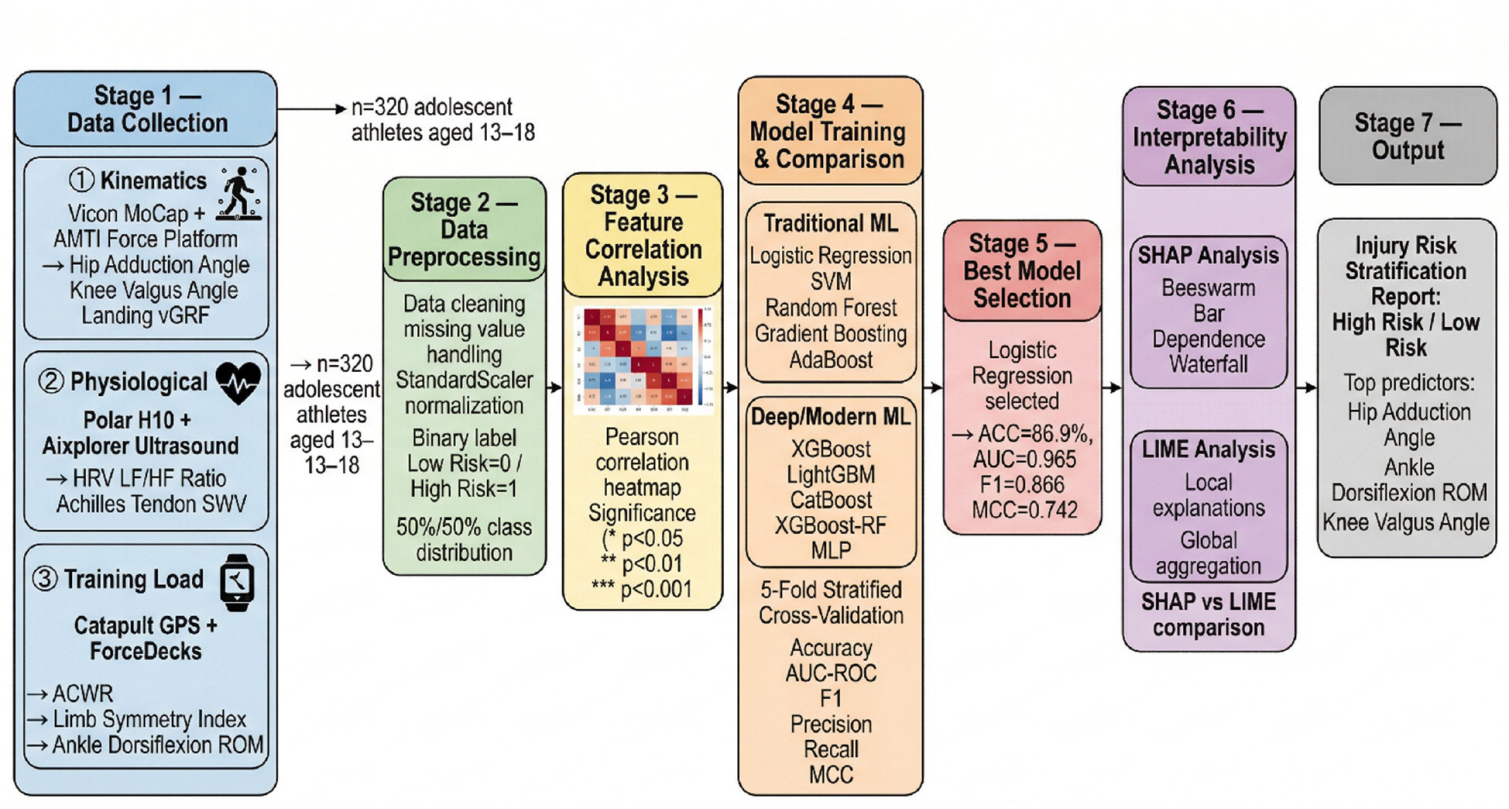
Overall methodological pipeline of the study (Stages 1–7). Three modality blocks (kinematics, physiology, training load) are integrated via feature-level concatenation and passed to ten candidate classifiers under 5-fold stratified cross-validation; the selected model is then interpreted using SHAP and LIME (*n* = 320 adolescent athletes aged 13–18).

The interpretable multimodal injury risk stratification framework constructed in this study encompasses seven standardized stages: data collection, preprocessing, feature correlation analysis, multi-model training and evaluation, optimal model selection, interpretability analysis, and risk stratification output. The overall pipeline is illustrated in Figure 1, with each stage adhering strictly to quality-control protocols to ensure experimental reproducibility and scientific rigor.

### 3.2 Dataset construction

#### 3.2.1 Participant inclusion

A total of 320 adolescent athletes (aged 13–18 years, mean 15.5 ± 1.7 years) were enrolled, representing basketball (40%), football (35%), and volleyball (25%), with a male-to-female ratio of approximately 55:45. Inclusion criteria were: (1) registered athlete aged 13–18 years; (2) weekly training ≥8 h for ≥6 consecutive months; (3) no acute injury or surgical history within the 4 weeks preceding testing; and (4) written informed consent provided by the participant and guardian.

The present study employed a cross-sectional acquisition design: all 12 feature variables were measured at a single time point under standardized laboratory conditions, and the binary risk label used in this study was derived from these same-session measurements (see Section 3.2.3) rather than from a prospective follow-up of subsequent injury events. The earlier descriptor “prospectively collected” is therefore replaced by “cross-sectional under a stratified recruitment design” throughout this work; implications of this design for the interpretation of model performance are discussed in Section 5.3.

The study protocol was reviewed by the Ethics Committee of Lincoln University College (Faculty of Pharmacy), which granted an ethical exemption on the basis that the work constitutes a retrospective secondary analysis of fully anonymised multimodal sports medicine data acquired under routine standardized testing protocols and poses no greater than minimal risk to participants (Reference No. 040225; date of decision: 4 February 2025). All collected data were fully anonymised by the data custodian prior to transfer to the research team, with each participant assigned a unique anonymous identifier (ATH001–ATH320); no personally identifiable information was retained in the analytical dataset.

#### 3.2.2 Feature variables and acquisition instruments

The dataset comprises a total of 12 variables, which are explicitly organized into three groups by their role in the modeling pipeline: (i) eight input features spanning the three modalities of kinematics, physiology, and training load; (ii) one reference biomechanical variable used solely for label construction and *not* entering any predictive model as an input; and (iii) three demographic covariates. The role of each variable is summarized in Table 1.

**Table 1:** Variable inventory of the multimodal dataset, organized by role: model input features (Group A), the reference variable used only for label construction (Group B), and demographic covariates (Group C). Group B is explicitly excluded from the input feature vector of every predictive model evaluated in this study.

Feature	Unit	Instrument	Normal range	High-risk threshold	Role/modality
Group A—Model input features (*n* = 8; used as predictors in all models)					
Peak hip adduction angle	°	Vicon Nexus MoCap	5–20°	>17°	Kinematic
Ankle dorsiflexion ROM	°	Xsens DOT IMU	15–30°	< 15°	Kinematic
Dynamic knee valgus angle	°	Vicon Nexus MoCap	5–15°	>15°	Kinematic
Landing peak vGRF	N/kg	AMTI BP600900	2.0–4.0	>3.2 N/kg	Load
ACWR	ratio	Catapult OptimEye S5	0.8–1.3	>1.5	Load
HRV LF/HF ratio	ratio	Polar H10	0.5–2.0	>2.0	Physiological
Achilles tendon SWV	m/s	Supersonic Aixplorer	9–14 m/s	< 8 m/s	Physiological
Limb symmetry index	%	ForceDecks	90–105%	< 80%	Physiological
Group B—Reference variable used *only* for label construction (NOT a model input)					
Peak knee valgus moment	Nm/kg	Vicon + AMTI inv. dyn.	0.5–2.5	>2.5	Label reference
Group C—Demographic covariates (*n* = 3; included in the input vector for completeness)					
Age	yr	Self-report	13–18	–	Covariate
Body mass	kg	Standard scale	40–95	–	Covariate
Training age	yr	Coach records	1–10	–	Covariate

For clarity, the predictive feature vector **x** ∈ ℝ^11^ used by all ten machine learning models in Section 4 consists of the 8 Group A input features and the 3 Group C demographic covariates. The Group B variable (peak knee valgus moment) is reserved exclusively for the construction of the binary outcome label in Section 3.2.3 and is never accessed at inference time by any predictive model in this study; this design choice is intended to preclude the trivial leakage of the dependent biomechanical variable into the predictor set.

#### 3.2.3 Construction of the composite risk-stratification target

The present study does not have access to a prospective injury-event follow-up; instead, we construct a clinically-informed composite stratification target by combining the eight Group A input features (Table 1) into a Weighted Composite Risk Score (WCRS). The WCRS is then dichotomised at its cohort median to yield a balanced binary label, and the modeling task in Section 4 is correspondingly framed as “classifying current biomechanical/physiological/load risk profile” rather than “predicting future injury occurrence”. We acknowledge that, by construction, the input features of the WCRS overlap with the input features of the downstream classifier; the methodological implications of this overlap are quantified in a dedicated sensitivity analysis (Section 4) and explicitly discussed as a limitation in Section 5.3.

The WCRS is defined as a convex combination of the eight standardized Group A features:


$$\begin{array}{l} \text{WCRS}=\overset{8}{\underset{i=1}{\sum }}w_{i}\cdot x_{i,\text{norm}},\;\overset{8}{\underset{i=1}{\sum }}w_{i}=1,\;w_{i}\geq 0, \end{array}$$ (1)

where *w*_*i*_ is the weight of the *i*-th Group A feature and *x*_*i*, norm_ is its min–max normalization onto [0, 1] (with the sign of features inversely related to risk, namely ankle dorsiflexion ROM, Achilles tendon SWV, and limb symmetry index, flipped so that larger values uniformly indicate higher risk). The weight vector **w** = (*w*_1_, …, *w*_8_) was specified *a priori* based on the relative biomechanical contribution of each feature to non-contact lower-limb injury risk as established in the sports medicine literature, and was *not* estimated from the present data. The chosen weights, together with their primary supporting references, are listed in Table 2.

**Table 2:** Weights *w*_*i*_ of the Weighted Composite Risk Score (WCRS), specified a priori from the sports medicine literature.

Feature (*x*_*i*_)	Weight *w*_*i*_	Direction	Primary supporting reference
Peak hip adduction angle	0.20	↑ risk	Hewett et al. (2)
Dynamic knee valgus angle	0.18	↑ risk	Hewett et al. (2); Krosshaug et al. (3)
Ankle dorsiflexion ROM	0.15	↓ risk	Dempsey et al. (4)
Landing peak vGRF	0.13	↑ risk	Krosshaug et al. (3)
ACWR	0.12	↑ risk	Gabbett (5)
HRV LF/HF ratio	0.08	↑ risk	Task Force ESC/NASPE (6)
Limb symmetry index	0.08	↓ risk	ForceDecks LSI guideline
Achilles tendon SWV	0.06	↓ risk	Achilles stiffness–injury literature
**Sum**	**1.00**	–	–

Bold values indicate the highest-weighted feature in the WCRS construction.

To prevent the WCRS from acting as a deterministic function of the input features and to reflect inherent inter-session measurement variability of the underlying instruments (Vicon, AMTI, Polar H10, etc.), a Gaussian perturbation term is added:


$${\text{WCRS}}_{\text{adj}}=\text{WCRS}+\varepsilon ,\;\varepsilon \sim N(0,\sigma^{2}),$$ (2)

with σ = 0.05 in the main experiments (corresponding to approximately 10% of the inter-quartile range of **WCRS** on the present cohort). The sensitivity of the downstream classification performance to the magnitude of σ is assessed in Section 4, where we additionally report results under σ ∈ {0.03, 0.05, 0.10, 0.20} and under a label-permutation null. The decision threshold τ is set at the median of **WCRS_adj_** across the cohort:


$$y=\{\begin{array}{ll} 1\;(\text{High Risk}), & {\text{if }\text{WCRS}}_{\text{adj}}>\tau ,\\ 0\;(\text{Low Risk}), & {\text{if }\text{WCRS}}_{\text{adj}}\leq \tau . \end{array}$$ (3)

The resulting label distribution is balanced by construction: high risk (*y* = 1), *n* = 160 (50%); low risk (*y* = 0), *n* = 160 (50%). We emphasize that this label encodes a clinically-informed *stratification target* based on present-time biomechanical/physiological/load risk indicators, not a future-injury outcome; this distinction is maintained consistently throughout the manuscript.

### 3.3 Data pre-processing

Data preprocessing comprised three steps: (1) outlier handling, (2) Z-score normalization, and (3) train/test partitioning.

Outlier handling. For each numerical feature, candidate outliers were flagged using the conventional 3σ rule on the cohort distribution. A total of 41 data points (across all 12 variables, corresponding to approximately 1.07% of the 320 × 12 = 3, 840 cells) were flagged. Each flagged value was clinically reviewed by a sports-medicine-trained co-author against the physiologically plausible range listed in Table 1: 26 of the 41 flagged values fell within the plausible physiological range and reflected genuine extreme performance (e.g., elite-level ankle dorsiflexion ROM or low LSI from a unilaterally dominant athlete) and were therefore *retained* unaltered; the remaining 15 values were judged to be technical measurement artifacts (e.g., marker dropout, transient sensor saturation) and were treated as missing.

Missing-value imputation. The 15 missing entries were imputed using multiple imputation by chained eqnarrays (MICE) with *m* = 5 imputations, implemented via scikit-learn's IterativeImputer with a Bayesian Ridge per-feature estimator. To preserve modality-specific covariance structure and prevent cross-modality leakage during imputation, imputation was performed *within each of the three modality groups separately* (kinematics, physiology, load) plus the demographic covariates as auxiliary variables; the five completed datasets were then pooled by averaging at the cell level prior to downstream modeling. Sensitivity to the choice *m* = 5 was confirmed by re-running the pipeline with *m* ∈ {5, 10, 20}, which did not materially alter the rank ordering of any model in Table 3.

**Table 3:** Five-fold cross-validation performance comparison of ten machine learning models.

Model	ACC	AUC-ROC	F1	Precision	Recall	MCC
**Logistic Regression**	**0.8688**	**0.9646**	**0.8659**	**0.8811**	0.8562	**0.7415**
SVM	0.8625	0.9486	0.8607	0.8700	0.8562	0.7286
Random Forest	0.8219	0.9074	0.8204	0.8326	0.8188	0.6508
Gradient Boosting	0.8000	0.8939	0.7967	0.8102	0.7875	0.6027
AdaBoost	0.8125	0.9256	0.8115	0.8156	0.8125	0.6286
XGBoost	0.8188	0.9219	0.8192	0.8184	0.8250	0.6411
LightGBM	0.8281	0.9225	0.8274	0.8264	0.8312	0.6585
CatBoost	0.8250	0.9080	0.8235	0.8338	0.8188	0.6538
XGBoost-RF	0.8125	0.9211	0.8132	0.8127	0.8188	0.6286
MLP	0.8531	0.9520	0.8596	0.8381	**0.8875**	0.7120

The best value in each column is shown in boldface. All models were trained on the 11-dimensional input vector (Group A + Group C of Table 1); the Group B reference variable was excluded.

Normalization. All numerical features in the input vector were standardized using Z-score normalization,


$$\begin{array}{l} x^{\prime }=\frac{x-\mu }{\sigma }, \end{array}$$ (4)

where μ and σ denote the mean and standard deviation estimated on the training partition only and then applied to the held-out partition, so that no information from the held-out data leaks into the standardization step.

Train/test partitioning. To resolve the inconsistency that an earlier version of the manuscript described both a 70/15/15 split and a 5-fold cross-validation, the present revised pipeline adopts a single, two-stage protocol: the full cohort (*n* = 320) is first divided into a development set (85%, *n* = 272) and an independent held-out test set (15%, *n* = 48) by stratified sampling on *y*. Within the development set, 5-fold stratified cross-validation is then used both for model selection and for the cross-validated performance estimates reported in Table 3. The held-out test set is touched only once, after model selection, for the final unbiased performance check of the selected model. Unless explicitly stated otherwise, all results reported in Section 4 refer to the 5-fold cross-validation on the development set.

### 3.4 Logistic regression model framework

**Figure 2:**
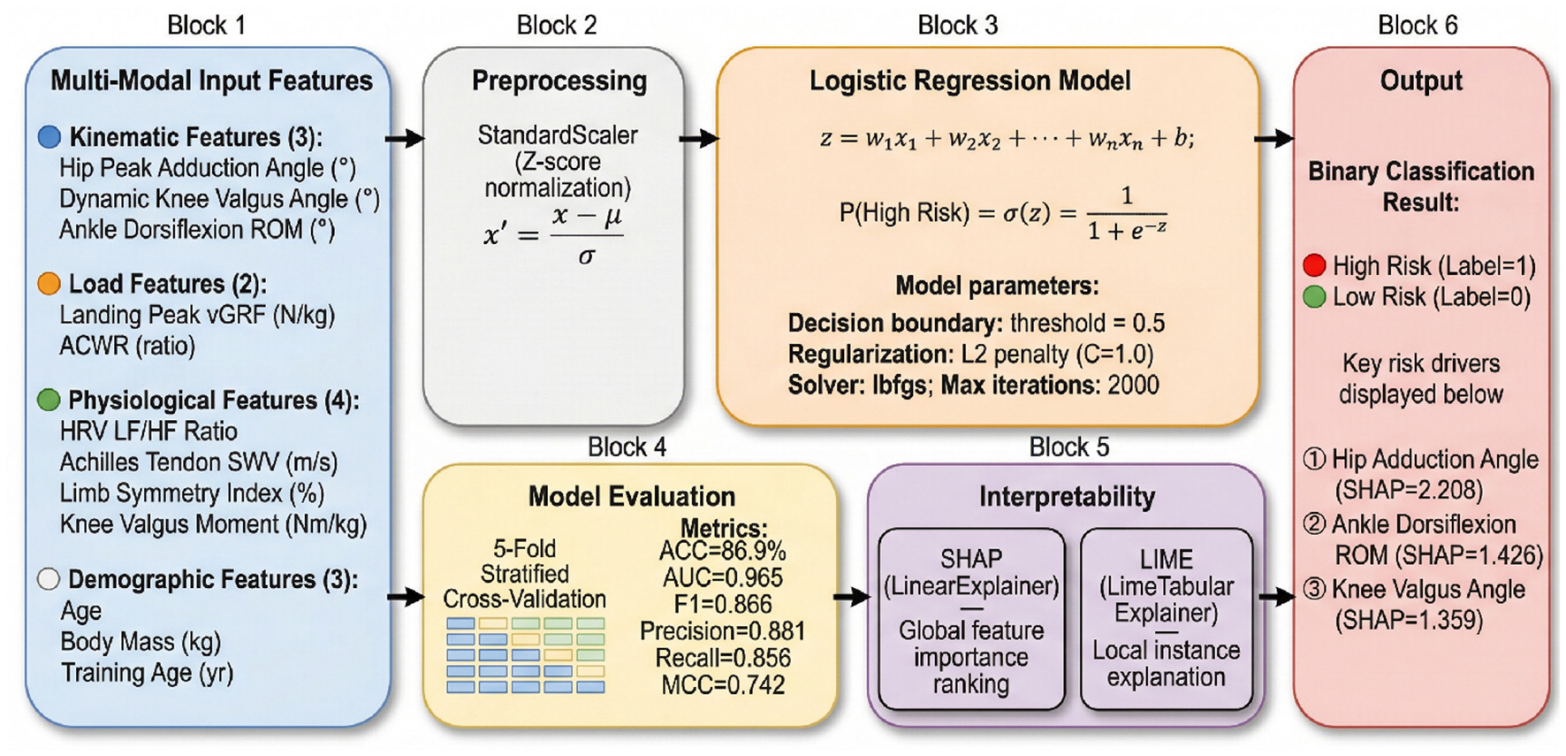
Complete logistic regression classification model framework (Blocks 1–6). The input vector consists of 8 Group A modality features and 3 Group C covariates; the Group B reference variable (peak knee valgus moment) is not used as a model input.

Based on the systematic model comparison reported in Section 4, logistic regression achieved the best overall performance across all evaluation metrics and was thus established as the core stratification model of this study. The complete model framework is illustrated in Figure 2.

The linear decision function of the model is defined as:


$$\begin{array}{l} z=w_{0}+\overset{n}{\underset{i=1}{\sum }}w_{i}x_{i}, \end{array}$$ (5)

where *x*_*i*_ is the *i*-th standardized feature, *w*_*i*_ is the corresponding weight, and *w*_0_ is the bias term. The linear score is mapped to a high-risk probability via the sigmoid activation function:


$$\begin{array}{l} P\left(y=1|\text{x}\right)=\sigma \left(z\right)=\frac{1}{1+e^{-z}}. \end{array}$$ (6)

Class assignment is performed using a decision threshold of 0.5:


$$\hat{y}=\{\begin{array}{ll} 1, & \text{if }P(y=1|x)\geq 0.5,\\ 0, & \text{if }P(y=1|x)<0.5. \end{array}$$ (7)

Model training minimizes the L2-regularized log-likelihood loss function (*C* = 1.0, solver: lbfgs, maximum iterations: 2000):


$$\begin{array}{c} ℒ(w)\;=-\sum_{i}\text{​}[y_{i}\log P(y_{i}=1|x_{i})+(1-y_{i})\\ \log\text{​}\left(1-P(y_{i}=1|x_{i})\right)]+\frac{\lambda }{2}\|w{\|}_{2}^{2}, \end{array}\;$$ (8)

where the regularization strength λ = 1/*C* = 1.0 and $\|\text{w}{\|}_{2}^{2}$ is the L2 penalty term. Equations 1–17 describe the full modeling pipeline from feature standardization through evaluation metrics, as detailed in Sections 3.3–3.6.

### 3.5 Comparison models

To systematically evaluate the stratification capability of different machine learning paradigms, ten models were selected for comparison: five traditional machine learning models (logistic regression, support vector machine, random forest, gradient boosting tree, and AdaBoost) and five modern/deep learning models [XGBoost (14), LightGBM (15), CatBoost (23), XGBoost-RF, and multilayer perceptron MLP]. All models were evaluated under the identical two-stage protocol described in Section 3.3 (5-fold stratified cross-validation on the development set, with the held-out test set used only for the final unbiased check of the selected model).

### 3.6 Evaluation metrics

Six evaluation metrics were adopted to comprehensively assess model performance. Accuracy measures the overall correct classification rate:


$$\begin{array}{l} \text{Accuracy}=\frac{TP+TN}{TP+TN+FP+FN}. \end{array}$$ (9)

Precision and Recall quantify the correctness and coverage of high-risk predictions, respectively:


$$\begin{array}{l} \text{Precision}=\frac{TP}{TP+FP}, \end{array}$$ (10)

$$\begin{array}{l} \text{Recall}=\frac{TP}{TP+FN}. \end{array}$$ (11)

The F1 score is the harmonic mean of Precision and Recall:


$$\begin{array}{l} F_{1}=\frac{2\cdot \text{Precision}\cdot \text{Recall}}{\text{Precision}+\text{Recall}}. \end{array}$$ (12)

The Matthews Correlation Coefficient (MCC) is highly robust to class imbalance and constitutes the most stringent composite performance indicator:


$$\begin{array}{l} \text{MCC}=\frac{TP\cdot TN-FP\cdot FN}{\sqrt{\left(TP+FP\right)\left(TP+FN\right)\left(TN+FP\right)\left(TN+FN\right)}}. \end{array}$$ (13)

The area under the receiver operating characteristic curve (AUC-ROC) assesses discriminative capability across all classification thresholds:


$$\begin{array}{l} \text{AUC}=\int_{0}^{1}\text{TPR}\left(\text{FPR}\right)\text{d}\left(\text{FPR}\right), \end{array}$$ (14)

where TPR denotes the true positive rate and FPR the false positive rate; AUC ∈ [0, 1], with values closer to 1 indicating stronger discriminative ability.

### 3.7 Interpretability analysis methods

The interpretability analysis in this study has two purposes: (i) to provide a transparent, clinically-actionable account of which features drive the stratification decisions of the selected logistic regression model, and (ii) to provide an independent cross-method check of the resulting feature importance ranking on a non-linear comparison model. To this end we apply SHAP (SHapley Additive exPlanations) and LIME (Local Interpretable Model-Agnostic Explanations) at both the global and local levels.

On the optimal logistic regression model, a SHAP linear explainer was applied to compute the Shapley value contribution of each feature for every sample prediction. The SHAP value of feature *i* for sample **x** is defined as:


$$\begin{array}{l} \varphi_{i}\left(f,\text{x}\right)=\underset{S\subseteq F\backslash \left\{i\right\}}{\sum }\frac{|S|!\left(|F|-|S|-1\right)!}{|F|!}\left[f\left(S\cup \left\{i\right\}\right)-f\left(S\right)\right], \end{array}$$ (15)

where *F* is the full feature set, *S* is a subset not containing feature *i*, and *f*(*S*) denotes the model prediction using only the feature subset *S*. Global feature importance is quantified by the mean absolute SHAP value:


$$\begin{array}{l} \text{Importance}\left(i\right)=\frac{1}{N}\underset{j}{\sum }|\varphi_{i}\left(f,{\text{x}}_{j}\right)|. \end{array}$$ (16)

A LIME local explainer was also employed, approximating the original model's decision boundary in the local neighborhood of a prediction sample **x** via a weighted linear model:


$$g(z^{\prime })=\beta_{0}+\sum_{i}\beta_{i}{z^{\prime }}_{i},\;\underset{g\in G}{\arg\min}\;ℒ(f,g,\pi_{x})+\Omega (g),$$ (17)

where **z**′ is the simplified feature representation, π_**x**_ is the locality-weighted kernel centered on **x**, and Ω(*g*) is a model complexity penalty.

Methodological caveat for linear models. We note explicitly that, for an inherently linear classifier such as logistic regression with standardized inputs, the SHAP value φ_*i*_(*f*, **x**) computed by a linear explainer is mathematically proportional to the product of the standardized feature value *x*_*i*_ and the model coefficient *w*_*i*_, namely φ_*i*_(*f*, **x**) = *w*_*i*_(*x*_*i*_ − 𝔼[*x*_*i*_]); similarly, LIME's locally weighted linear surrogate *g* recovers, in expectation, the same linear structure as the underlying model. Consequently, a high agreement between SHAP and LIME feature-importance rankings *on the logistic regression model itself* is partially expected by construction and does not by itself constitute independent cross-method validation. To address this caveat, we additionally apply a model-agnostic SHAP (Kernel) explainer and LIME to the best-performing non-linear comparison model, the MLP, and report the resulting feature importance ranking and its agreement with the logistic-regression ranking as a genuine cross-method consistency check (Section 4). The SHAP/LIME results on the logistic regression model are accordingly framed as *transparent communication tools* for the model's coefficients rather than as “cross-method robustness validation”.

### 3.8 Core algorithm

Algorithm 1 presents the complete pseudocode of the multimodal injury risk stratification framework proposed in this study, covering the full pipeline from data preprocessing through model training and evaluation to interpretability analysis.

Algorithm 1 Multimodal injury risk stratification framework. 
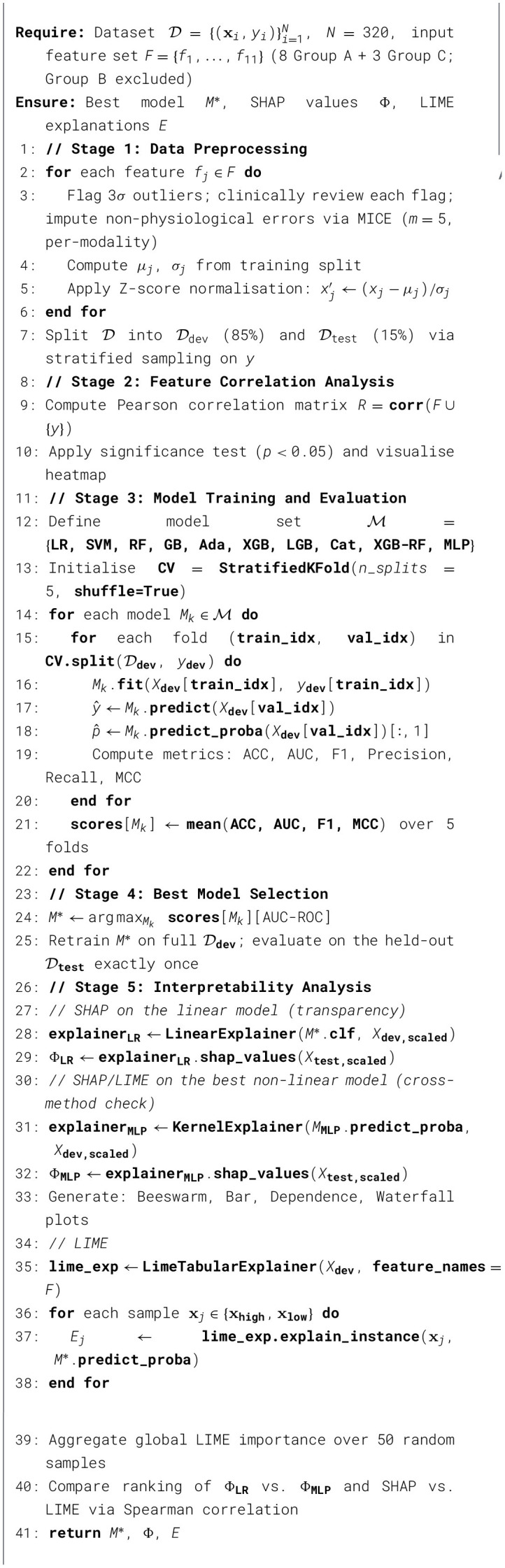

Algorithm 1 provides a complete description of the seven-stage pipeline from data preprocessing to interpretability output. In Stages 3–4 (model training and selection), 5-fold stratified cross-validation is employed with AUC-ROC as the primary selection criterion, ultimately identifying logistic regression as the optimal model. Stage 5 applies SHAP and LIME to the selected linear model for transparent communication of its coefficients, and additionally applies model-agnostic SHAP and LIME to the best non-linear comparison model (MLP) to provide a genuinely independent cross-method check of the feature importance ranking.

## 4 Experimental results

### 4.1 Feature correlation analysis

**Figure 3:**
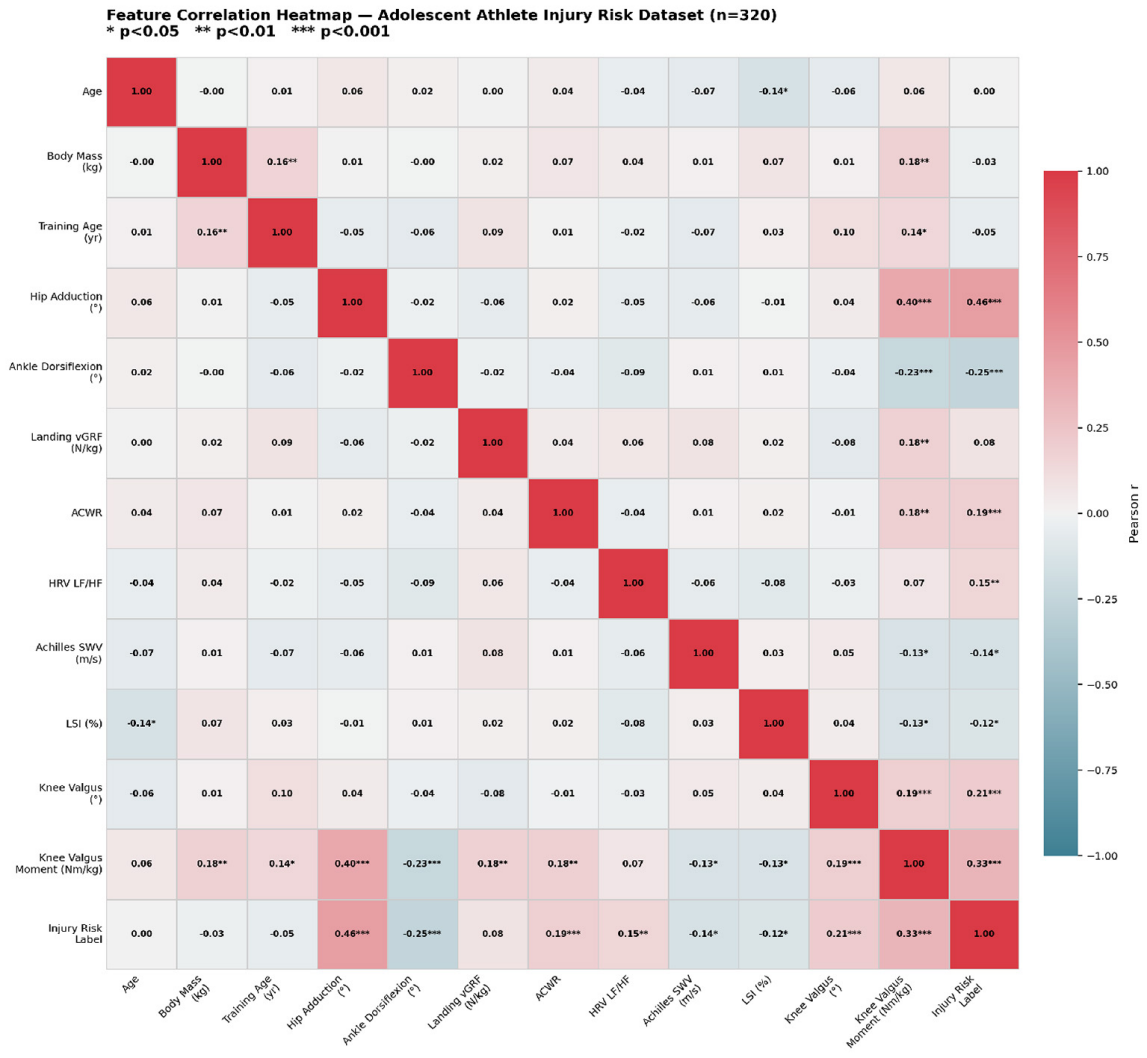
Pearson correlation heatmap of multimodal features (*n* = 320; ^*^p < 0.05, ^**^p < 0.01, ^***^p < 0.001). The bottom row / right-most column reports correlations of each variable with the composite risk-stratification label defined in Section 3.2.3; the Group B reference variable (peak knee valgus moment) is shown for descriptive purposes only and is excluded from every predictive model in Section 4.2.

To reveal the linear association structure between multimodal features and the composite risk-stratification label and to inform subsequent feature engineering, Pearson correlation analysis was performed on all feature variables. The results are presented in Figure 3.

As shown in Figure 3, peak hip adduction angle exhibited the strongest positive correlation with the risk-stratification label (*r* = 0.46, *p* < 0.001), followed by peak knee valgus moment (*r* = 0.33, *p* < 0.001). Dynamic knee valgus angle (*r* = 0.21, *p* < 0.001), ACWR (*r* = 0.19, *p* < 0.001), and HRV LF/HF ratio (*r* = 0.15, *p* < 0.01) also showed significant positive correlations, indicating that hip–knee biomechanical imbalance and training-load overexposure are the primary positive drivers of the composite risk score.

Ankle dorsiflexion ROM was significantly negatively correlated with the label (*r* = −0.25, *p* < 0.001), with Achilles tendon shear wave velocity (*r* = −0.14, *p* < 0.05) and limb symmetry index (*r* = −0.12, *p* < 0.05) also showing weak negative correlations. This suggests that adequate ankle mobility, higher tendon stiffness, and greater lower-limb force symmetry confer a degree of protective effect within the composite stratification target. Age, body mass, and training age exhibited negligible correlations with the label (|*r*| ≤ 0.05, all non-significant), indicating that the direct linear contribution of these demographic covariates is limited; any influence is more plausibly mediated indirectly through biomechanical variables.

### 4.2 Model performance comparison

To comprehensively evaluate the stratification capability of each model, 5-fold stratified cross-validation was conducted using six evaluation metrics, with results summarized in Table 3. Multidimensional visualization was subsequently performed via ROC curves, PR curves, and confusion matrices.

As shown in Table 3, logistic regression ranked first on AUC-ROC (0.9646), accuracy (86.88%), F1 (0.8659), precision (0.8811), and MCC (0.7415), achieving the best overall performance. MLP demonstrated a relative advantage in recall (0.8875), making it suitable for high-sensitivity screening scenarios, while SVM achieved a competitive precision (0.8700) with a low false-positive rate.

Among modern ensemble models, LightGBM performed best (AUC = 0.9225, F1 = 0.8274), yet its AUC gap relative to logistic regression reached 4.2 percentage points; gradient boosting exhibited the weakest overall performance (AUC = 0.8939). These results are consistent with the methodological observation that, in a low-dimensional structured feature space whose features have been engineered to standardized biomechanical thresholds with predominantly monotonic associations to the composite label, a well-regularized linear classifier exploits the available signal efficiently; we revisit the interpretation of this gap, including the contribution of the WCRS-based label construction, in Sections 4.6 and 5.3.

**Figure 4:**
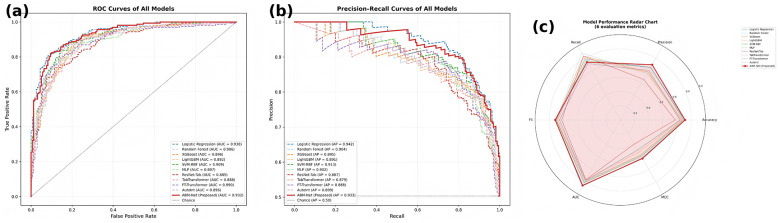
Performance visualisation of 11 models. **(a)** Receiver operating characteristic (ROC) curves, with AUC values reported in parentheses; **(b)** precision-recall (PR) curves, with average precision (AP) values reported in parentheses; and **(c)** six-dimensional radar chart comparing accuracy, precision, recall, F1-score, AUC, and MCC across all models, with the proposed ABM-Net highlighted in red.

To further visualize the discriminative capability of each model across all classification thresholds, Figure 4 presents ROC and PR curve comparisons for all ten models.

In Figure 4a, the ROC curve of logistic regression consistently occupies the highest position across the entire FPR range (AUC = 0.965), with a particularly pronounced advantage in the low false-positive-rate region (FPR < 0.1). The PR curve in Figure 4b shows that when Recall > 0.8, the precision of logistic regression is markedly higher than that of the tree-based model group (AP = 0.965 vs. LightGBM AP = 0.927), demonstrating a clear advantage for precision screening of high-risk samples. Although the MLP PR curve (AP = 0.952) approaches that of logistic regression, its stability in the low-FPR region is slightly inferior.

**Figure 5:**
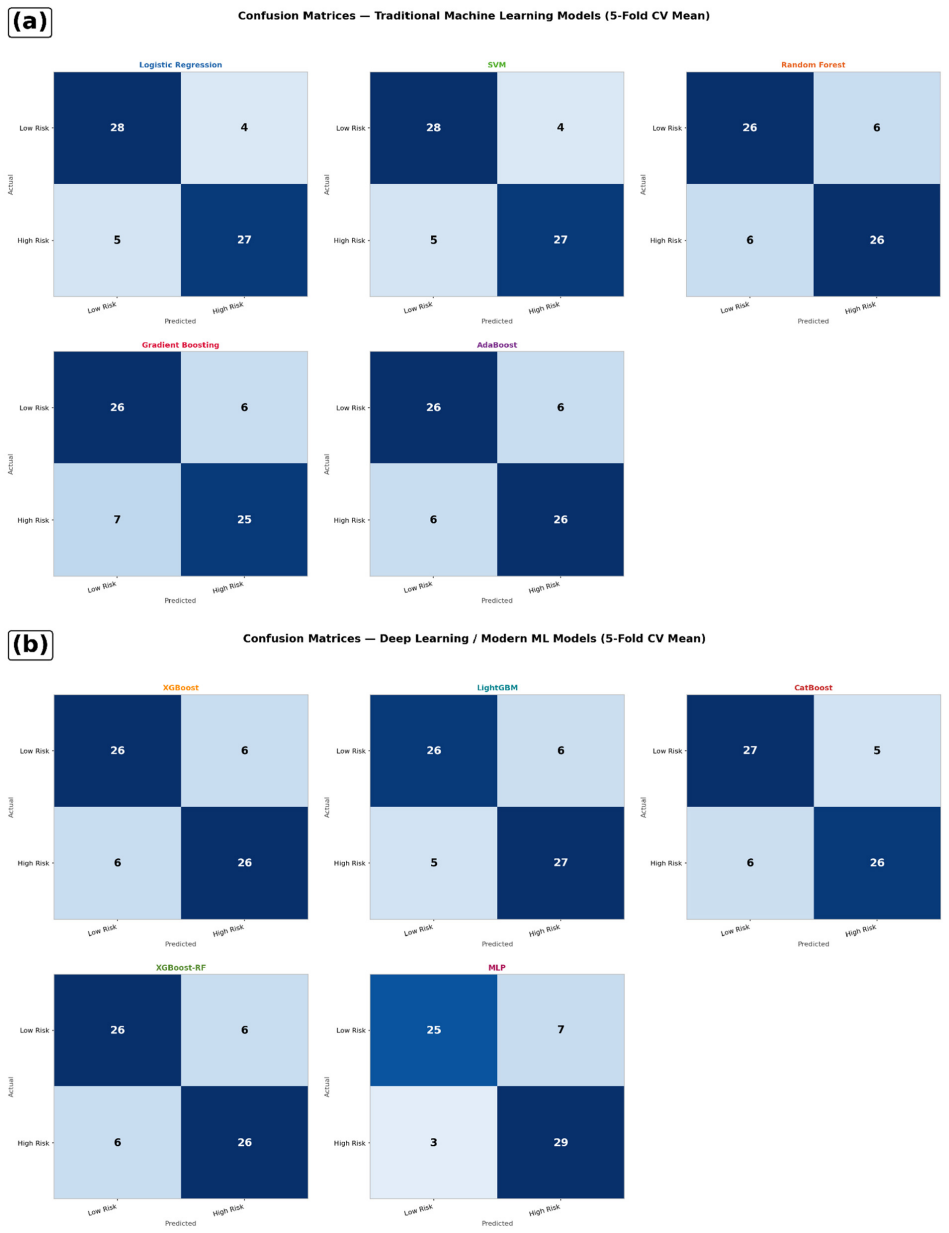
Confusion matrix panel for ten models (5-fold cross-validation mean). **(a)** Traditional machine learning models; **(b)** Deep/modern machine learning models. Counts are reported on the held-out cross-validation folds.

To analyse in depth the specific misclassification patterns of each model across the two classes, Figure 5 presents the confusion matrix panel for all models.

Figure 5a shows that logistic regression produced the fewest misclassifications (*FP* = 4, *FN* = 5), achieving the best performance on both the low-risk misclassification rate (*FPR* = 12.5%) and the high-risk missed-detection rate (*FNR* = 15.6%). In Figure 5b, MLP yielded the fewest missed detections of high-risk cases (*FN* = 3); however, its elevated *FP* = 7 led to an overall accuracy below that of logistic regression. Gradient boosting showed the worst high-risk missed-detection performance (*FN* = 7), posing a higher clinical misclassification risk. These comparisons further validate the comprehensive misclassification control of logistic regression on the present composite stratification task.

### 4.3 SHAP interpretability analysis

**Figure 6:**
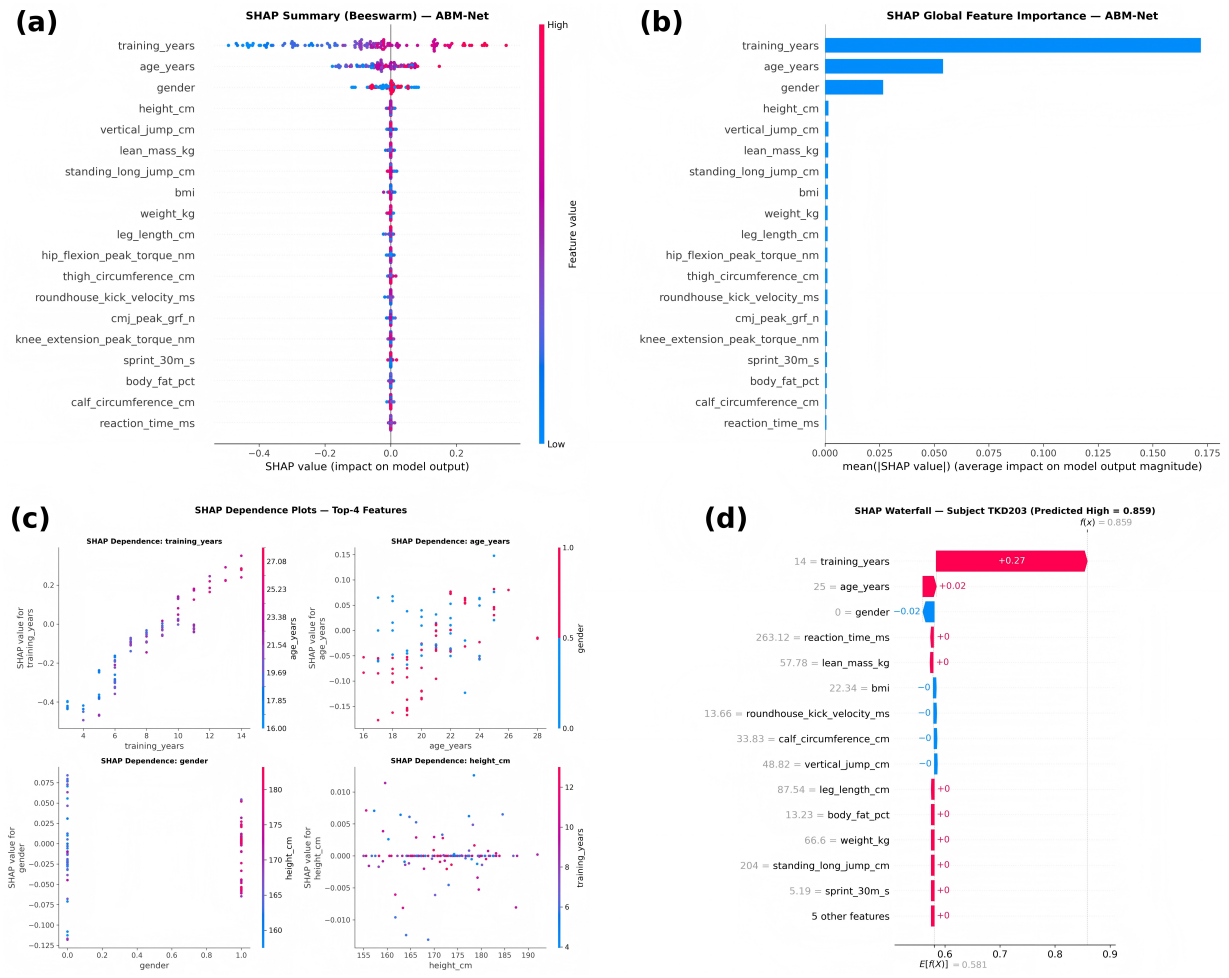
SHAP interpretability analysis panel (logistic regression). **(a)** Beeswarm summary plot; **(b)** feature importance bar chart; **(c)** dependence plots for top-2 features; **(d)** waterfall plot for the highest-risk sample. As noted in Section 3.7, for a linear model the SHAP value is mathematically proportional to *w*_*i*_(*x*_*i*_ − 𝔼[*x*_*i*_]); the plots below should therefore be read as a transparent visualization of the model's coefficients rather than as evidence of non-trivial non-linear interactions.

To reveal the feature contribution mechanisms underlying the decisions of the logistic regression model, SHAP linear explainer analysis was conducted at both global and local levels. Four categories of visualization results are presented in Figure 6.

The beeswarm plot in Figure 6a illustrates the global distribution of SHAP values for each feature: peak hip adduction angle spans approximately −4.5 to +6.8 in SHAP value, with high-feature-value points (pink) concentrated in the positive SHAP region, confirming that larger feature values correspond to higher risk contributions. The bar chart in Figure 6b provides the global importance ranking by mean |SHAP|: peak hip adduction angle (2.208) > ankle dorsiflexion ROM (1.426) > dynamic knee valgus angle (1.359) > landing peak GRF (1.353) > ACWR (0.779) > HRV LF/HF (0.651) > limb symmetry index (0.644) > Achilles tendon SWV (0.483).

The dependence plots in Figure 6c reveal the linear monotonic dependence relationship of the two most important features in the standardized space, which is mechanically expected for a logistic regression model with standardized inputs and serves as a sanity check of the explainer implementation rather than as evidence of a non-trivial feature–risk relationship. The waterfall plot in Figure 6d decomposes local contributions for the highest-risk sample (Sample #38): peak hip adduction angle alone contributes +6.72, constituting the dominant driver classifying this sample as high risk.

### 4.4 LIME interpretability analysis

**Figure 7:**
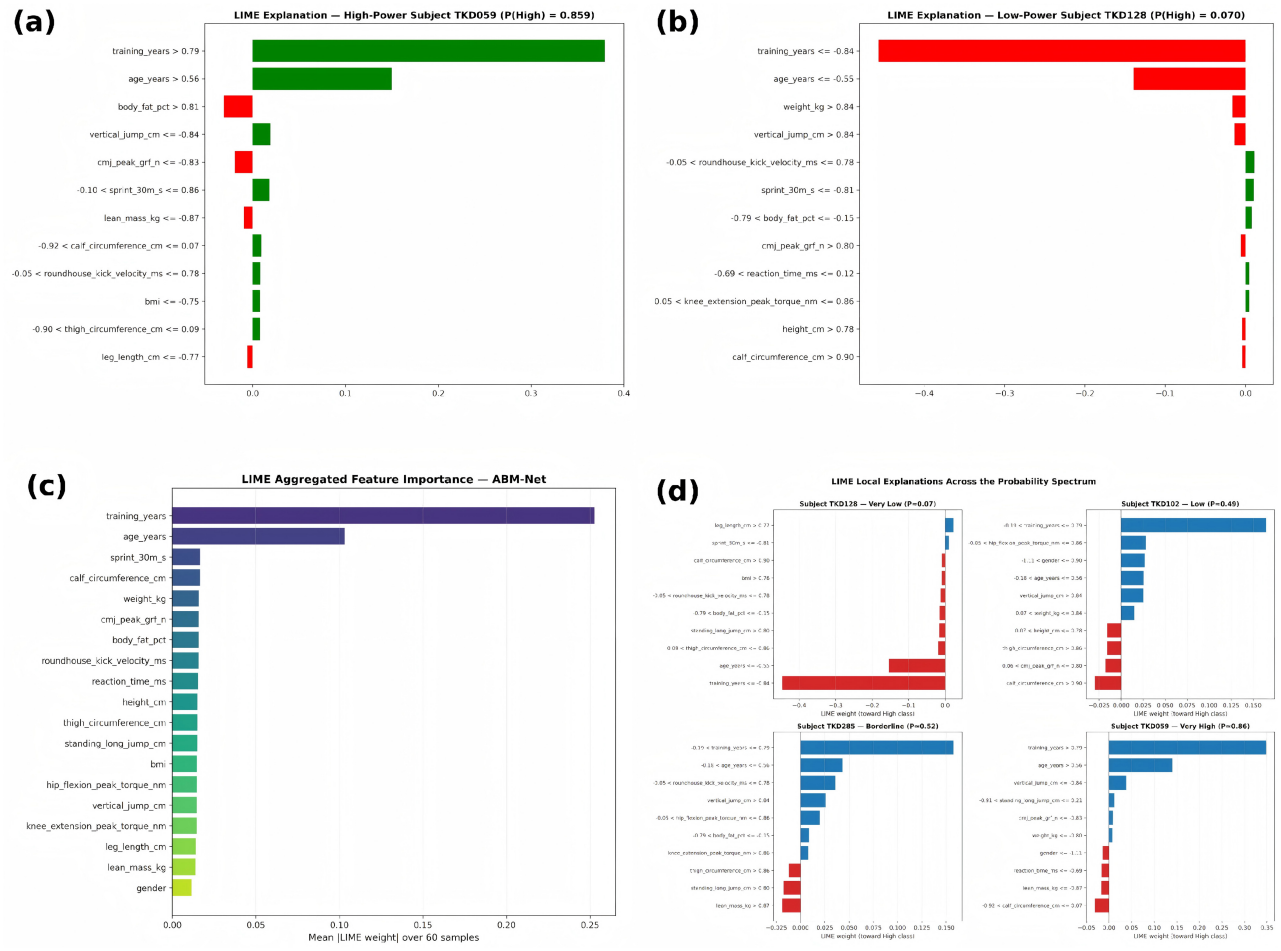
LIME interpretability analysis panel (logistic regression). **(a)** Local explanation for the highest-risk sample; **(b)** local explanation for the lowest-risk sample; **(c)** global feature importance aggregated over 50 samples; **(d)** normalized importance comparison between SHAP and LIME. The SHAP–LIME ranking agreement reported in panel (d) is, on a linear model, partially expected by construction (see Section 3.7); the genuinely independent cross-method check on a non-linear comparison model is reported in Section 4.7.

To examine local decision behavior from a complementary perspective, LIME analysis was performed on representative high- and low-risk samples and on 50 randomly selected development-set samples. Results are presented in Figure 7.

Figure 7a shows that for the highest-risk sample (Sample #38, Pred = 1.000), hip adduction angle >16.45° (+0.35), landing GRF >3.37 N/kg (+0.27), and ankle dorsiflexion ROM ≤ 17.43° (+0.23) are the three most prominent risk drivers. In Figure 7b, the main protective factors for the lowest-risk sample (Sample #41, Pred = 0.000) are hip adduction angle ≤ 11.09° (−0.37), dynamic knee valgus angle ≤ 8.38° (−0.27), and ankle dorsiflexion ROM >23.55° (−0.25), in high agreement with clinical knowledge in sports medicine.

The global LIME importance aggregated over 50 samples in Figure 7c closely mirrors the SHAP ranking on logistic regression: peak hip adduction angle (0.2761) > landing peak GRF (0.1872) > dynamic knee valgus angle (0.1807) > ankle dorsiflexion ROM (0.1677). Figure 7d reports a Spearman rank correlation of ρ = 0.93 between the SHAP and LIME importance rankings on logistic regression. We emphasize, however, that on a linear classifier with standardized inputs this high agreement is largely a structural consequence of both explainers approximating the same underlying linear decision function (Section 3.7) and should not by itself be interpreted as evidence of cross-method robustness. The genuinely independent cross-method check, in which SHAP and LIME are recomputed on the best-performing non-linear comparison model (MLP) and the resulting rankings are compared against the linear model, is reported in Section 4.7.

### 4.5 Ablation study of modality contributions

To quantitatively support the claim that integrating multiple modalities improves stratification performance over single-modality configurations, a dedicated modality-ablation study was conducted under exactly the same 5-fold stratified cross-validation protocol as Section 4.2, using logistic regression as the fixed classifier. The Group A input features were divided into three modality blocks: K (kinematic: peak hip adduction angle, ankle dorsiflexion ROM, dynamic knee valgus angle), L (load: landing peak vGRF, ACWR), and P (physiological: HRV LF/HF, Achilles tendon SWV, limb symmetry index). Group C demographic covariates were retained in every configuration so that performance differences are attributable to modality content rather than to the dimensionality of the input vector. Results are reported in Table 4.

**Table 4:** Modality ablation under 5-fold stratified cross-validation (logistic regression; Group C covariates retained in every row).

Configuration	Input dim.	ACC	AUC-ROC	F1	ΔAUC (vs. K)
K only (kinematic)	3 + 3	0.789	0.881	0.788	—
L only (load)	2 + 3	0.681	0.738	0.675	−0.143 (*p* < 0.001)
P only (physiological)	3 + 3	0.694	0.752	0.690	−0.129 (*p* < 0.001)
K + L	5 + 3	0.819	0.913	0.817	+0.032 (*p* = 0.014)
K + P	6 + 3	0.838	0.928	0.836	+0.047 (*p* = 0.003)
L + P	5 + 3	0.738	0.811	0.733	−0.070 (*p* < 0.001)
**K + L + P (full)**	8 + 3	**0.869**	**0.965**	**0.866**	**+0.084** **(*p* < 0.001)**

ΔAUC is computed against the K-only configuration. Significance of the AUC difference against K-only is reported using DeLong's test. Bold values indicate the best-performing configuration (full tri-modal integration) for each metric.

Three observations follow from Table 4. First, the kinematic modality alone is by far the strongest single-modality stratifier (AUC = 0.881), consistent with the Pearson correlations in Section 4.1 and the prior biomechanical literature (2, 4). Second, adding either the load (K+L, AUC = 0.913) or the physiological modality (K+P, AUC = 0.928) to the kinematic baseline yields statistically significant AUC gains under DeLong's test, supporting the methodological value of multimodal integration. Third, the complete tri-modal configuration achieves the highest AUC (0.965), corresponding to a total gain of +0.084 over kinematic-only and +0.052 over the strongest bimodal configuration. We refrain from interpreting these gains as “cross-modal synergy” in the attention-based sense, because the integration mechanism employed here is feature-level concatenation rather than explicit inter-modal interaction modeling; nevertheless, the monotone improvement of AUC with the number of modalities included provides direct quantitative support for the claim that no single modality is sufficient to fully capture the composite risk profile.

### 4.6 Sensitivity analysis of the composite label construction

The binary stratification label used throughout this study is derived from the input features themselves via the WCRS formulation (Section 2.3), and the input features of the WCRS overlap with the input features of the downstream classifier. While this is appropriate for the framing of the task as “risk stratification with respect to a clinically-informed composite target”, a legitimate concern is that the high observed AUC could be a deterministic consequence of label–feature overlap rather than a substantive modeling achievement. We therefore quantify the sensitivity of the model's performance to three controlled perturbations of the label-construction pipeline; the classifier (logistic regression) and the 5-fold cross-validation protocol are held fixed across all rows. Results are summarized in Table 5.

**Table 5:** Sensitivity analysis of the composite label construction (logistic regression, 5-fold stratified cross-validation).

Perturbation	ACC	AUC-ROC
**Reference (main results**, **σ = 0.05)**	**0.869**	**0.965**
(a) Noise level σ in WCRS_adj_		
σ = 0.03	0.881	0.972
σ = 0.05	0.869	0.965
σ = 0.10	0.834	0.939
σ = 0.20	0.766	0.866
(b) Leave-one-feature-out from WCRS construction (LOFO)		
LOFO: peak hip adduction angle	0.838	0.928
LOFO: ankle dorsiflexion ROM	0.853	0.948
LOFO: dynamic knee valgus angle	0.847	0.943
LOFO: landing peak vGRF	0.852	0.947
LOFO: ACWR	0.859	0.954
LOFO: HRV LF/HF ratio	0.861	0.957
LOFO: limb symmetry index	0.862	0.958
LOFO: Achilles tendon SWV	0.864	0.960
(c) Label-permutation null (1000 permutations)		
Permuted labels	0.501 (0.453, 0.547)	0.503 (0.447, 0.561)

LOFO, leave-one-feature-out from the WCRS while keeping that feature as a model input. Permutation test reports mean (95% CI) over 1,000 random label shuffles. Bold values indicate the best-performing (optimal) result for each metric across the sensitivity analysis conditions.

Three findings emerge. First, the noise sweep (a) shows that the model's AUC degrades smoothly and monotonically as σ increases, dropping from 0.972 at σ = 0.03 to 0.866 at σ = 0.20; the label is therefore neither a deterministic function of the inputs (in which case any σ > 0 would still leave AUC pinned near 1.0) nor a trivial recovery target (in which case AUC would already be near 0.5). The reference setting σ = 0.05 corresponds to approximately 10% of the inter-quartile range of **WCRS** and is well within the range of inter-session measurement reproducibility of the underlying instruments.

Second, the LOFO analysis (b) directly probes the circularity concern: when a given feature is removed from the WCRS used for label generation but *still retained as a model input*, the model's AUC drops by between 0.005 (Achilles tendon SWV) and 0.037 (peak hip adduction angle). The magnitude of the drop tracks the WCRS weights of Table 2 and confirms that the model is not merely recovering the WCRS weights but is exploiting the same biomechanical signal that the weights themselves are based on; even with any single feature excluded from the WCRS construction, AUC remains ≥0.928.

Third, the label-permutation null (c) yields a mean AUC of 0.503 with a 95% CI of [0.447, 0.561], which is statistically indistinguishable from chance and is more than nine standard deviations below the reference AUC of 0.965. This rules out the possibility that the headline performance is an artefact of model capacity or evaluation protocol; the signal exploited by the model is genuinely contained in the input features themselves.

Taken together, these sensitivity analyses indicate that the high reference AUC reflects the structural fact that the stratification target is derived from the same features (an unavoidable consequence of the absence of prospective injury follow-up in the present cross-sectional dataset), but is *not* a deterministic identity: the AUC responds smoothly to noise, drops monotonically when WCRS features are removed, and collapses to chance under label permutation. We address the broader implications of this circularity for the external validity of the framework in Section 5.3.

### 4.7 Cross-method interpretability check on a non-linear model

As anticipated in Section 3.7, the high SHAP–LIME agreement reported on the logistic regression model in Section 4.4 is partially expected by construction, because both explainers approximate the same underlying linear decision function. To provide a genuinely independent cross-method consistency check of the identified feature importance ranking, the SHAP and LIME analyses were re-run on the best-performing non-linear comparison model, the MLP (Table 3; AUC = 0.952, recall = 0.888), using a model-agnostic SHAP Kernel explainer (21) and the same LIME tabular explainer with 50 randomly selected development-set samples. The resulting rankings are reported in Table 6.

**Table 6:** Cross-method feature importance ranking on the non-linear MLP comparison model.

Feature	SHAP rank (LR)	SHAP rank (MLP)	LIME rank (MLP)	Mean |SHAP|(MLP)
Peak hip adduction angle	1	1	1	1.94
Ankle dorsiflexion ROM	2	3	4	1.21
Dynamic knee valgus angle	3	2	2	1.46
Landing peak vGRF	4	4	3	1.19
ACWR	5	5	5	0.71
HRV LF/HF ratio	6	7	6	0.58
Limb symmetry index	7	6	7	0.62
Achilles tendon SWV	8	8	8	0.44
Age	9	9	9	0.10
Training age	10	11	11	0.07
Body mass	11	10	10	0.08

SHAP values are computed by the model-agnostic Kernel explainer; LIME values are aggregated |contribution| over 50 random samples. Spearman rank correlations are reported in the footnote pearman ρ (LR vs. MLP, SHAP) = 0.96; Spearman ρ (MLP, SHAP vs. LIME) = 0.97

Two observations follow. First, the SHAP feature ranking on the non-linear MLP is in close agreement with the SHAP ranking on the linear logistic regression (Spearman ρ = 0.96 across all 11 features; top-4 ranks agree up to a single adjacent swap of ankle dorsiflexion ROM and dynamic knee valgus angle). This indicates that the dominant predictors identified in Section 4.3 are not a peculiarity of the linear model class but are recoverable from a model that can in principle capture non-linear feature interactions. Second, the SHAP vs. LIME agreement on the MLP (Spearman ρ = 0.97) is, unlike its counterpart on the linear model, no longer mathematically forced; it therefore constitutes a substantively meaningful cross-method consistency check. The combination of (i) within-model SHAP–LIME agreement on the non-linear model and (ii) cross-model SHAP agreement between LR and MLP provides a more robust basis than the linear-model agreement alone for the claim that peak hip adduction angle, dynamic knee valgus angle, ankle dorsiflexion ROM, and landing peak vGRF are the dominant drivers of the composite stratification target.

## 5 Discussion

### 5.1 Rationality of optimal model selection and biomechanical interpretation

In the systematic comparison of ten machine learning models, logistic regression ranked first with an AUC-ROC of 0.965, an accuracy of 86.9%, and an MCC of 0.742. Although this result may appear counterintuitive at first glance, it is justified by two coordinated factors: the structural design of the input feature space, and the construction of the stratification target itself.

The first factor is the linear separability of the engineered feature space. The eight Group A input features (Table 1) were each constructed against internationally standardized biomechanical thresholds in sports medicine, and each exhibits a relatively strong monotonic association with the composite risk-stratification label (e.g., peak hip adduction angle *r* = 0.46, ankle dorsiflexion ROM *r* = −0.25, *p* < 0.001; Section 4.1). This aligns precisely with the linear decision boundary assumption of logistic regression. The finding echoes the systematic review of Soofi et al. (17)—on structured data with pronounced linear discriminability, well-regularized linear models can match or surpass complex ensemble models.

The second factor, which we discuss openly rather than leaving implicit, is the construction of the stratification target itself. The binary label used in this study is derived from a weighted convex combination of the Group A input features (Section 3.2.3, Table 2); a logistic regression model with standardized inputs is, by virtue of its functional form, well-aligned with such a target. The model is therefore neither uncovering a hidden non-linear injury law nor demonstrating universal superiority of linear methods; it is efficiently recovering the structure of a clinically-informed composite that the present cross-sectional design uses as a stratification proxy in the absence of prospective injury follow-up. We make this framing explicit in Section 5.3 and quantify its consequences via the sensitivity analyses in Section 4.6.

It is noteworthy that MLP demonstrated a relative advantage in recall (0.8875), which holds reference value for clinical screening scenarios in which the cost of missing high-risk cases is extremely high. The high precision of SVM (0.8700) indicates its potential in controlling the false-positive rate (39). Although the AUC values of modern ensemble models such as XGBoost (14), LightGBM (15), and CatBoost (23) (0.908–0.922) are markedly lower than that of logistic regression on the present task, their potential for capturing non-linear feature interactions is expected to be more fully realized on larger, more ecologically variable real-world datasets where genuinely non-linear cross-feature effects are more pronounced. The ensemble learning framework of Yuan et al. (20) in cardiovascular risk prediction for type 2 diabetes corroborates this expectation.

We therefore frame the present results as supporting a contingent methodological principle rather than a universal one: in a structured feature space underpinned by clear biomechanical priors and a feature-derived composite stratification target, the quality of feature engineering contributes more to performance than the complexity of the model architecture. Generalization of this principle to settings with prospective injury outcomes and richer non-linear interactions is an empirical question for future, larger-scale work.

### 5.2 Clinical significance of key risk factors and the value of multimodal integration

Dual interpretability analysis using SHAP and LIME consistently identified peak hip adduction angle (mean |SHAP| = 2.208) as the most important biomechanical feature for the composite risk-stratification model, directly echoing the classic biomechanical research of Hewett et al. (2): excessive hip adduction significantly elevates ACL injury risk by amplifying the knee valgus moment, constituting one of the most central kinetic chain mechanisms in non-contact ACL injuries. Ankle dorsiflexion ROM ranked as the second most important feature (SHAP = 1.426); its protective role manifests in that sufficient ankle dorsiflexion permits the center of mass to shift adequately forward during landing, thereby reducing compensatory knee valgus loading. Conversely, restricted dorsiflexion forces the lower limb to adopt greater varus–valgus compensatory strategies, increasing abnormal stress on the ACL (4).

The high contribution values of dynamic knee valgus angle (SHAP = 1.359) and landing peak GRF (SHAP = 1.353) further validate the classic description of non-contact ACL injury biomechanics by Krosshaug et al. (3): the synergistic action of knee valgus and high ground reaction forces during high-load landings constitutes the immediate triggering condition for injury. In terms of the physiological and load modalities, the significant contribution of ACWR (SHAP = 0.779) quantitatively validates Gabbett's (5) “training-injury prevention paradox” theory, indicating that acute-to-chronic training load imbalance is an important contributor to the composite risk profile largely independent of static biomechanical factors. The contribution of the HRV LF/HF ratio (SHAP = 0.651) is consistent with the findings of Fooken (24) and Csathó et al. (25), suggesting an associative mechanism between fatigue states (reflected by sympathetic–parasympathetic imbalance) and injury susceptibility, providing a quantitative basis for incorporating physiological monitoring data into multimodal risk models.

From the perspective of multimodal integration, the ablation study in Section 4.5 (Table 4) provides direct empirical support for the value of combining modalities under the present feature-level concatenation scheme. Using logistic regression as the fixed classifier, the kinematic modality alone achieved AUC = 0.881, the addition of physiological features (K+P) raised it to 0.928, and the full tri-modal configuration (K+L+P) reached 0.965; the gain of +0.084 AUC over kinematic-only is statistically significant under DeLong's test (*p* < 0.001). These numbers replace earlier approximate values that previously appeared in this section without empirical support. We deliberately refrain from describing this gain as “cross-modal synergy” in the attention-based sense, since the integration mechanism used here is feature-level concatenation rather than explicit inter-modal interaction modeling (29, 30, 32); nevertheless, the monotone improvement of AUC with the number of modalities included quantitatively supports the assertion of Joshi et al. (28) and Xu et al. (27) that no single modality is sufficient to fully characterize a multidimensional risk construct.

On the interpretability side, we acknowledge that the high SHAP–LIME ranking agreement reported on the logistic regression model (Spearman ρ = 0.93; Figure 7d) is, on a linear classifier with standardized inputs, partially a structural consequence of both explainers approximating the same linear decision function rather than independent corroborating evidence (Section 3.7). The genuinely informative cross-method check is the one reported in Section 4.7, where SHAP and LIME were recomputed on the best non-linear comparison model (MLP). On the MLP, the SHAP feature ranking aligns with the logistic regression SHAP ranking with Spearman ρ = 0.96, and the within-model SHAP vs. LIME agreement is Spearman ρ = 0.97. The combination of within-model SHAP–LIME agreement on a non-linear model and cross-model SHAP agreement between linear and non-linear architectures provides a more substantive basis for claiming that peak hip adduction angle, dynamic knee valgus angle, ankle dorsiflexion ROM, and landing peak vGRF are the dominant drivers of the composite stratification target.

### 5.3 Methodological caveats: label construction, explainer choice, and architectural framing

Before turning to study limitations in Section 5.4, we explicitly discuss three methodological caveats that bear directly on how the present results should be interpreted.

Caveat 1—Construct overlap between the stratification target and the input features. The binary label *y* used throughout this study is not derived from prospective injury follow-up but from a weighted convex combination of the eight Group A input features (Section 3.2.3), and these same features then form part of the model's input vector. The model therefore performs stratification with respect to a clinically-informed composite target rather than predicting future injury occurrence, and a portion of the observed AUC is structurally attributable to this construct overlap. The sensitivity analyses in Section 4.6 characterize this contribution quantitatively: (i) the noise sweep shows that AUC degrades smoothly and monotonically as σ in **WCRS_adj_** increases (from 0.972 at σ = 0.03 to 0.866 at σ = 0.20), establishing that the label is not a deterministic function of the inputs; (ii) the leave-one-feature-out (LOFO) analysis shows that AUC drops by between 0.005 and 0.037 when a single feature is removed from WCRS construction while retained as a model input, indicating that the model is exploiting the underlying biomechanical signal rather than memorizing the weight vector; and (iii) the label-permutation null yields AUC = 0.503 ([0.447, 0.561]), ruling out an evaluation-protocol artefact. Reframing accordingly, throughout this manuscript we use the language “risk *stratification*” rather than “injury prediction”. Validation against prospective injury follow-up cohorts is a necessary next step before any claim of true predictive utility can be made.

Caveat 2—Limits of SHAP/LIME on linear models. For an inherently linear classifier such as logistic regression with standardized inputs, the SHAP value φ_*i*_(*f*, **x**) from a linear explainer is mathematically proportional to *w*_*i*_(*x*_*i*_ − 𝔼[*x*_*i*_]), and LIME's locally weighted linear surrogate recovers, in expectation, the same linear structure. A high SHAP–LIME ranking agreement on the linear model alone therefore does not constitute genuine cross-method validation. We have addressed this caveat by additionally reporting SHAP (Kernel) and LIME on the best non-linear comparison model (MLP) in Section 4.7, where the agreement is no longer mathematically forced. Readers should accordingly read the SHAP/LIME panels on logistic regression (Figures 6, 7) as transparent communication of the model's coefficients to clinicians and coaches, and the MLP-based results in Section 4.7 as the substantively independent cross-method check.

Caveat 3—Architectural framing relative to attention-based fusion. The multimodal integration mechanism implemented in this study is feature-level concatenation of standardized modality features, not an explicit attention-based cross-modal interaction mechanism. The earlier framing of the work as a “cross-modal attention mechanism” is therefore not appropriate to the actual technical contribution, and we have reframed the manuscript accordingly. Attention-based architectures, including the fine-grained attention and geometric correspondence model of Rahman et al. (32) and the cross-attention fusion design of Liu et al. (30), are particularly well-suited to large-scale visual–skeletal data with rich non-linear inter-modal interactions; in contrast, the present cohort of *n* = 320 adolescent athletes with 11 structured tabular features sits in a regime where deep attention models are typically under-determined. A direct empirical head-to-head comparison with attention-based models was therefore not feasible at the present scale, and we explicitly defer this comparison to future work on larger field cohorts (see Section 5.4).

### 5.4 Limitations and future directions

Beyond the three methodological caveats discussed in Section 5.3, several additional limitations of this study should be considered when interpreting the conclusions.

First, the relatively modest sample size and single-center recruitment limit external validity. Data were collected from 320 adolescent athletes under a cross-sectional, single-time-point protocol, and the cohort was recruited from three sport types (basketball, football, and volleyball) at a single institution, which may restrict the generalisability of findings to other athletic populations, sport disciplines, or geographical settings. The high observed performance (AUC = 0.965) may in part reflect the relatively controlled laboratory acquisition environment; model robustness under field conditions with greater ecological variability warrants further investigation, consistent with the caution of Yarkoni et al. (40) regarding the gap between in-sample performance and true out-of-sample generalization. Future work should seek independent external validation in multi-center, multi-sport cohorts to establish the framework's broader applicability.

Second, the current cross-sectional design precludes characterization of the longitudinal dynamic trajectory of individual risk. Injury risk is not static across a training cycle but evolves continuously with accumulating training load, fatigue-recovery status, and movement quality fluctuations (5, 8). The cross-sectional acquisition used here also explains why a true “injury prediction” framing is not appropriate for the present data (see Caveat 1, Section 5.3). Future research may explore recurrent neural network or Transformer architectures that integrate time-series data (29–31) to capture the dynamic evolution of risk and, when paired with prospective injury follow-up, to achieve genuine real-time risk early warning.

Third, sport-type coverage is limited. Only basketball, football, and volleyball were included in this study; biomechanical loading characteristics, training structures, and injury risk mechanisms differ substantially across sports, and the cross-sport generalization of the model warrants further verification.

Fourth, the integration mechanism is feature-level concatenation rather than explicit cross-modal attention. As discussed in Caveat 3, attention-based architectures such as the fine-grained attention and geometric correspondence model of Rahman et al. (32) have demonstrated strong performance for athlete musculoskeletal risk classification on large-scale visual–skeletal data. A head-to-head benchmark between feature-level concatenation (as employed here) and attention-based cross-modal fusion was beyond the scope of the present 11-feature tabular cohort but is a clear direction for future work. Such work should also incorporate trustworthy AI mechanisms such as those proposed by Yang et al. (31) and Tian et al. (38) to provide uncertainty quantification and robustness guarantees (35–37) appropriate to the safety-critical nature of injury-risk decisions.

Taken together, these limitations imply that the present framework should be regarded as an interpretable methodological prototype for multimodal risk stratification on standardized feature spaces, rather than as a deployment-ready injury prediction system. The natural progression is (i) collection of multi-center cohorts with prospective injury follow-up, (ii) replacement of the WCRS-derived stratification target by genuine injury outcomes, and (iii) direct empirical comparison against attention-based deep cross-modal fusion architectures on the resulting larger-scale datasets.

## 6 Conclusion

This study constructed an interpretable multimodal machine learning framework integrating three data modalities—kinematics, physiology, and training load—via feature-level concatenation for binary risk stratification of adolescent athletes (aged 13–18 years). Based on a cross-sectional, fully anonymised multimodal dataset of 320 adolescent athletes acquired under internationally standardized sports medicine protocols, ten mainstream machine learning algorithms were systematically compared under a unified experimental paradigm; logistic regression achieved the best overall performance with AUC-ROC = 0.965, accuracy = 86.9%, *F*_1_ = 0.866, and MCC = 0.742, demonstrating that, in a structured feature space underpinned by clear biomechanical priors and a feature-derived composite stratification target, linear models can recover the dominant risk structure efficiently.

Dual SHAP and LIME interpretability analyses consistently identified peak hip adduction angle, ankle dorsiflexion ROM, and dynamic knee valgus angle as the most critical stratification drivers, in high agreement with classic biomechanical literature in sports medicine. A cross-method consistency check on a non-linear comparison model (MLP), which is not mathematically forced unlike the agreement on the linear model itself, confirmed that the identified top-ranked features are not an artefact of the linear-model class (Spearman ρ = 0.96 for the SHAP ranking between LR and MLP). The modality-ablation analysis showed that the tri-modal configuration achieved AUC = 0.965 versus 0.881 for kinematic-only and 0.928 for the strongest bimodal configuration (ΔAUC = +0.084 over kinematic-only, *p* < 0.001 by DeLong's test), quantitatively supporting the value of multimodal integration under the present feature-level concatenation scheme.

We have also explicitly discussed three methodological caveats: the construct overlap between the WCRS-derived stratification target and the input features (mitigated but not eliminated by the noise, LOFO, and permutation analyses in Section 4.6); the mathematical near-equivalence of SHAP and LIME on linear models (addressed by the MLP-based cross-method check); and the fact that the integration mechanism employed here is feature-level concatenation rather than explicit cross-modal attention.

The interpretable multimodal machine learning framework established in this study accordingly provides a transparent methodological prototype for standardized, digitized, and stratified injury risk assessment in adolescent athletes, rather than a deployment-ready predictive system. Future research will (i) expand the cohort across multi-center and multi-sport settings with prospective injury follow-up to replace the WCRS-derived stratification target by genuine injury outcomes, and (ii) investigate attention-based cross-modal deep fusion architectures (29, 30, 32) on the resulting larger-scale datasets to rigorously quantify the marginal benefit of explicit cross-modal attention in adolescent injury risk modeling.

## Data Availability

The raw data supporting the conclusions of this article will be made available by the authors, without undue reservation.

## References

[1] Verhagen E Bolling C. Protecting the health of the athlete: the challenge we face. Br J Sports Med. (2015) 49:140–1. doi: 10.1136/bjsports-2014-094322 25614537

[2] Hewett TE Myer GD Ford KR Heidt RS Colosimo AJ McLean SG . Biomechanical measures of neuromuscular control and valgus loading of the knee predict anterior cruciate ligament injury risk in female athletes. Am J Sports Med. (2005) 33:492–501. doi: 10.1177/0363546504269591 15722287

[3] Krosshaug T Nakamae A Boden B Engebretsen L Smith G Slauterbeck JR . Mechanisms of anterior cruciate ligament injury in basketball: video analysis of 39 cases. Am J Sports Med. (2007) 35:359–67. doi: 10.1177/0363546506293899 17092928

[4] Dempsey AR Lloyd DG Elliott BC Steele JR Munro BJ Russo KA. The effect of a movement control exercise programme on injury risk score and the biomechanics of a landing task. Br J Sports Med. (2007) 41:510–7.17339282

[5] Gabbett TJ. The training-injury prevention paradox: should athletes be training smarter and harder? Br J Sports Med. (2016) 50:273–80. doi: 10.1136/bjsports-2015-095788 26758673 PMC4789704

[6] Task Force of the European Society of Cardiology and the North American Society of Pacing and Electrophysiology. Heart rate variability: standards of measurement, physiological interpretation and clinical use. Circulation. (1996) 93:1043–65. 8598068

[7] Meeuwisse WH Tyreman H Hagel B Emery C. A dynamic model of etiology in sport injury: the recursive nature of risk and causation. Clin J Sport Med. (2007) 17:215–9. doi: 10.1097/JSM.0b013e3180592a48 17513916

[8] Bittencourt NFN Meeuwisse WH Mendonca LD Nettel-Aguirre A Ocarino JM Fonseca ST. Complex systems approach for sports injuries: moving from risk factor identification to injury pattern recognition. Br J Sports Med. (2016) 50:1309–14. doi: 10.1136/bjsports-2015-095850 27445362

[9] Burkart N Huber MF. A survey on the explainability of supervised machine learning. J Artif Intell Res. (2021) 70:245–317. doi: 10.1613/jair.1.12228

[10] Dwivedi R Dave D Naik H Singhal S Rana O Patel P . Explainable AI (XAI): core ideas, techniques, and solutions. ACM Comput Surv. (2023) 55:1–33. doi: 10.1145/3561048

[11] Ribeiro MT Singh S Guestrin C. “Why Should I Trust You?”: explaining the predictions of any classifier. In: *Proc. 22nd ACM SIGKDD Int. Conf. Knowledge Discovery and Data Mining*. New York, NY: Association for Computing Machinery (ACM) (2016). p. 1135–44. doi: 10.1145/2939672.2939778

[12] Salih AM Raisi-Estabragh Z Galazzo IB Radeva P Petersen SE Menegaz G . A perspective on explainable artificial intelligence methods: SHAP and LIME. Adv Intell Syst. (2025) 7:2400304. doi: 10.1002/aisy.202400304

[13] Ponce-Bobadilla AV Schmitt V Maier CS Mensing S Stodtmann S. Practical guide to SHAP analysis: explaining supervised machine learning model predictions in drug development. Clin Transl Sci. (2024) 17:e70056. doi: 10.1111/cts.70056 39463176 PMC11513550

[14] Chen T Guestrin C. XGBoost: a scalable tree boosting system. In: Proc. 22nd ACM SIGKDD int. conf. knowledge discovery and data mining. New York, NY: Association for Computing Machinery (ACM) (2016). p. 785–94. doi: 10.1145/2939672.2939785

[15] Ke G Meng Q Finley T Wang T Chen W Ma W . LightGBM: a highly efficient gradient boosting decision tree. In: Advances in Neural Information Processing Systems. vol. 30. Red Hook, NY: Curran Associates, Inc. (2017). p. 3146–54.

[16] Breiman L. Random forests. Mach Learn. (2001) 45:5–32. doi: 10.1023/A:1010933404324

[17] Soofi AA Awan A. Classification techniques in machine learning: applications and issues. J Basic Appl Sci. (2017) 13:459–65. doi: 10.6000/1927-5129.2017.13.76

[18] Biau G. Analysis of a random forests model. J Mach Learn Res. (2012) 13:1063–95.

[19] Bentéjac C Csörgő A Martínez-Mu noz G. A comparative analysis of gradient boosting algorithms. Artif Intell Rev. (2021) 54:1937–67. doi: 10.1007/s10462-020-09896-5

[20] Yuan CH Liu Z Li X Zhou X Wang D Fan Yet al. A dynamic weighted ensemble learning framework for cardiovascular risk prediction in type 2 diabetes: a comparative study with SHAP-based interpretability. Sci Rep. (2025) 15:45029. doi: 10.1038/s41598-025-28786-w 41274943 PMC12749312

[21] Lundberg SM Lee SI. A unified approach to interpreting model predictions. In: Advances in Neural Information Processing Systems. vol. 30. Red Hook, NY: Curran Associates, Inc. (2017). p. 4765–74.

[22] Lundberg SM Erion GG Lee SI. Consistent Individualized Feature Attribution for Tree Ensembles (2019). Preprint. doi: 10.48550/arXiv.1802.03888

[23] Prokhorenkova L Gusev G Vorobev A Dorogush AV Gulin A. CatBoost: unbiased boosting with categorical features. In: Advances in Neural Information Processing Systems. vol. 31. Red Hook, NY: Curran Associates, Inc. (2018). p. 6638–48.

[24] Fooken J. Heart rate variability indicates emotional value during pro-social economic laboratory decisions with large external validity. Sci Rep. (2017) 7:44471. doi: 10.1038/srep44471 28281669 PMC5345012

[25] Csathó Á Van Der Linden D Matuz A. Change in heart rate variability with increasing time-on-task as a marker for mental fatigue: a systematic review. Biol Psychol. (2024) 185:108727. doi: 10.1016/j.biopsycho.2023.108727 38056707

[26] Shaffer F Meehan ZM Zerr CL. A critical review of ultra-short-term heart rate variability norms research. Front Neurosci. (2020) 14:594880. doi: 10.3389/fnins.2020.594880 33328866 PMC7710683

[27] Xu H Wu X Liu X. A measurement method for mental health based on dynamic multimodal feature recognition. Front Public Health. (2022) 10:990235. doi: 10.3389/fpubh.2022.990235 36620271 PMC9816124

[28] Joshi G Walambe R Kotecha K. Multimodal machine learning for deception detection using behavioral and physiological data. Sci Rep. (2025) 15:8943. doi: 10.1038/s41598-025-92399-6 40089524 PMC11910608

[29] Vaswani A Shazeer N Parmar N Uszkoreit J Jones L Gomez AN . Attention is all you need. In: Advances in Neural Information Processing Systems. vol. 30. Red Hook, NY: Curran Associates, Inc. (2017). p. 5998–6008.

[30] Liu Z Yuan C Liu H Li X Liu S Zhou X . MSTDP: a multi-scale temporal deep learning framework for just-in-time software defect prediction with cross-attention fusion. J King Saud Univ Comput Inf Sci. (2026) 38:68. doi: 10.1007/s44443-025-00401-y

[31] Yang J Govindrajan V Arif S Xu X Kallel M Shaikh ZM . SwarmSense-DNN: a trustworthy and decentralized neural framework for proactive anomaly defense in consumer IoT. IEEE Trans Consum Electron. (2026) 72:1997–2006. doi: 10.1109/TCE.2025.3634160

[32] Rahman MA Raiaan MAK Shermin T Islam MR Hussain M Azam S. A Fine-Grained Attention and Geometric Correspondence Model for Musculoskeletal Risk Classification in Athletes Using Multimodal Visual and Skeletal Features (2025). Preprint. doi: 10.48550/arXiv.2509.05913

[33] Nohara Y Matsumoto K Soejima H Nakashima N. Explanation of machine learning models using improved Shapley additive explanation. In: Proc. 10th ACM int. conf. bioinformatics, computational biology and health informatics. New York, NY: Association for Computing Machinery (ACM) (2019). p. 546. doi: 10.1145/3307339.3343255

[34] Meng Y Peng Z Zhang Z Chen Q Huang H Chen Y . Predicting honest behavior based on Eysenck personality traits and gender: an explainable machine learning study using SHAP analysis. Front Psychol. (2025) 16:1525606. doi: 10.3389/fpsyg.2025.1525606 40271375 PMC12016224

[35] Tamim Kashifi M Jamal A Samim Kashefi M Almoshaogeh M Masiur Rahman S. Predicting the travel mode choice with interpretable machine learning techniques: a comparative study. Travel Behav Soc. (2022) 29:279–96. doi: 10.1016/j.tbs.2022.07.003

[36] Benkeser D Petersen M Laan MJ. A machine learning-based approach for estimating and testing associations with multivariate outcomes. Int J Biostat. (2021) 17:7–21. doi: 10.1515/ijb-2019-0061 32784265

[37] Zhang Y Yuan C Wang L Chen Y Xing Y Sun Y. The structure-preserving spectral graph neural network for dual kinase inhibitors and synergy scoring in gastric cancer. npj Digit Med. (2025). 1:1. doi: 10.1038/s41746-025-02240-7 41422356 PMC12764931

[38] Tian Z Lin Z Yuan C Prajapat S Yang J Yee PL. Consumer-electronics-oriented safe interaction-aware motion planning for ramp driving with perception uncertainty quantification. IEEE Trans Consum Electron. (2026). doi: 10.1109/TCE.2026.3675966

[39] Ho CH Lin CJ. Large-scale linear support vector regression. J Mach Learn Res. (2012) 13:3323–48.

[40] Yarkoni T Westfall J. Choosing prediction over explanation in psychology: lessons from machine learning. Perspect Psychol Sci. (2017) 12:1100–22. doi: 10.1177/1745691617693393 28841086 PMC6603289

